# Association of two Common Single Nucleotide Polymorphisms (+45T/G and +276G/T) of *ADIPOQ* Gene with Coronary Artery Disease in Type 2 Diabetic Patients

**DOI:** 10.7508/ibj.2016.03.004

**Published:** 2016-07

**Authors:** Ghorban Mohammadzadeh, Mohammad-Ali Ghaffari, Habib Heibar, Mohammad Bazyar

**Affiliations:** 1 Hyperlipidemia Research Center, Dept. of Clinical Biochemistry, Faculty of Medicine, Ahvaz Jundishapur University of Medical Sciences, Ahvaz, Iran;; 2 Cellular and Molecular Research Center, Department of Clinical Biochemistry, Faculty of Medicine, Ahvaz Jundishapur University of Medical Sciences, Ahvaz, Iran;; 3 Cardiovascular Research Center, Dept. of Cardiology, Faculty of Medicine, Ahvaz Jundishapur University of Medical Sciences, Ahvaz, Iran;; 4 Department of Clinical Biochemistry, Faculty of Medicine, Ahvaz Jundishapur University of Medical Sciences, Ahvaz, Iran

**Keywords:** Adiponectin, Type 2 diabetes, Cronary artery disease, Single nucleotide polymorphisms

## Abstract

**Background:**

Adiponectin, an adipocyte-secreted hormone, is known to have anti-atherogenic, anti-inflammatory, and anti-diabetic properties. In the present study, the association between two common single nucleotide polymorphisms (SNPs) (+45T/G and +276G/T) of *ADIOPQ* gene and coronary artery disease (CAD) was assessed in the subjects with type 2 diabetes (T2DM).

**Methods:**

Genotypes of two SNPs were determined by polymerase chain reaction-restriction fragment length polymorphism in 200 subjects with T2DM (100 subjects with CAD and 100 without CAD).

**Results:**

The frequency of TT genotype of +276G/T was significantly elevated in CAD compared to controls (χ^2^=7.967, *P*=0.019). A similar difference was found in the allele frequency of +276G/T between two groups (χ^2^=3.895, *P*=0.048). The increased risk of CAD was associated with +276 TT genotype when compared to reference GG genotype (OR=5.158; 95% CI=1.016-26.182, *P*=0.048). However, no similar difference was found in genotype and allele frequencies of SNP +45T/G between two groups. There was a CAD protective haplotype combination of +276 wild-type and +45 mutant-type allele (276G-45G) (OR=0.37, 95% CI=0.16-0.86, *P*=0.022) in the subject population.

**Conclusion:**

Our findings indicated that T allele of SNP +276G/T is more associated with the increased risk of CAD in subjects with T2DM. Also, a haplotype combination of +45G/+276G of these two SNPs has a protective effect on the risk of CAD.

## INTRODUCTION

The incidence of coronary artery disease (CAD) is two to four times more frequent in diabetic patients than to non-diabetic subjects, which indicates the major cause of mortality and morbidity in this population^[^^1^^]^. The circulating levels of adiponectin, an adipocyte-derived hormone, have been demonstrated to be decreased in patients with the phenotypes of metabolic syndrome^[^^2^^]^, including obesity^[^^3^^]^, type 2 diabetes (T2DM)^[^^4^^]^, and insulin resistance^[^^5^^]^. Furthermore, it has been shown that low plasma levels of adiponectin are also correlated with CAD^[^^6^^,^^7^^]^. 

The adiponectin-coding gene, *ADIPOQ*, is located on chromosome 3q27, a genomic region identified as a susceptibility locus for the metabolic syndrome, T2DM and CAD through genome-wide scans^[^^8^^,^^9^^]^. The effects of two common single nucleotide polymorphisms (SNPs) of *ADIPOQ* gene, T/G substitution in exon 2 (+45T/G) and G/T substitution in intron 2 (+276G/T), on cardiovascular disease have been investigated in Caucasian and Korean populations^[^^9^^,^^10^^]^. The G allele at the +45T/G polymorphism has been shown to be associated with higher serum adiponectin concentrations^[^^11^^,^^12^^]^ and to improve insulin sensitivity^[^^13^^]^. 

A study has reported the protective effect of the G allele at the +45T/G polymorphism on the risk of CAD in European populations^[^^14^^]^. On the other hand, results from another study conducted on Italian population^[^^15^^]^ demonstrated that +276G/T polymorphism is associated with CAD risk in diabetic patients. In Asia, +276G/T polymorphism is not only correlated with T2DM^[^^16^^]^ and cardiovascular disease in Japanese patients^[^^17^^]^ but also with the metabolic syndrome in Korean subjects^[^^18^^]^. However, this variant was not correlated with T2DM or insulin resistance in another Korean population^[^^19^^]^. Therefore, it is likely that due to differences in clinical or ethnic backgrounds of the studied populations, the results of previous *ADIPOQ* genetic association studies are contradictory^[^^14^^-^^19^^]^. In addition, little is known about the association between two common SNPs of the *ADIPOQ* gene and the risk of CAD in Iranian subjects with T2DM. The aim of the current study was to investigate the association between two common SNPs (+45T/G and +276G/T) of the *ADIPOQ* gene and CAD in Iranian subjects with T2DM. 

## MATERIALS AND METHODS

**Patients**

In this study, 200 subjects with T2DM were selected based on American Diabetes Association (ADA) and divided into two groups: 100 patients with CAD (CAD cases) and 100 patients without CAD (controls). All patients enrolled in the study were Iranians with ancestry in the Khuzestan Province, Iran. The carotid angiography of all patients was carried out at the Department of Cardiology, Ahvaz Golestan Hospital (Ahvaz, Iran). All the recorded data of T2DM patients, who agreed to participate in the study, were reviewed. Criteria for the selection of T2DM and CAD patients have been described elsewhere^[^^20^^]^. The selected criterion for CAD, confirmed by coronary angiography, was ≥50% stenosis of at least one segment of a major coronary artery or its main branches. The control group consisted of T2DM patients aged over 35 years old, who had normal exercise tolerance test and negative history of CAD. Smokers, alcoholism, pregnant individuals, subjects under insulin therapy, and those with chronic liver and renal disease as well as acute infectious diseases, such as active diabetic foot, were not included. Demographic parameters of the subjects, including age, gender, duration of diabetes, height, weight, BMI, systolic and diastolic blood pressures were measured as described previously^[^^20^^]^. Serum levels of glucose, total cholesterol, HDL cholesterol and triglyceride were measured by enzymatic methods. Friedewald's formula^[^^21^^]^ was used to calculate the level of LDL cholesterol. Homeostasis model assessment of insulin resistance was used to determine insulin resistance as well as fasting serum insulin, and adiponectin was measured by ELISA as previously described elsewhere^[^^20^^]^. The study was approved by the Clinical Research Ethics Committee of Ahvaz Jundishapur University of Medical Sciences, and all subjects gave a written informed consent. Venous blood samples from all patients were collected in EDTA-containing tubes following an overnight fasting of at least 12 h.

**Genotyping the +45T/G and +276G/T polymor-phisms of** ***ADIPOQ*** **gene**

Genomic DNA was extracted from the whole blood using a DNA purification kit (SinaClon BioScience Co., Tehran, Iran). *ADIPOQ* +45T/G and +276G/T polymorphisms were determined using polymerase chain reaction-restriction fragment-length polymorphism analysis. The DNA fragment containing each SNP was amplified using the following primers: 

+45T/G (rs 2241766): 

5'-GCA GCT CCT AGA AGT AGA CTC TG-3' (forward) and 5′-TCT GTG ATG AAA GAG GCC AG-3' (reverse)

+276G/T (rs 1501299): 

5′-GTC TCT CCA TGG CTG ACA GT-3' (forward) and 5′- GGT GAA GAT GGG AAA GGG GA-3' (reverse)

The amplification reaction was performed in a 20-µl volume containing 12.5 µl commercially available PCR premix (AccuPower PCR PremiX; Bioneer, Daejeon, Korea), 2.0 µl (10 pmol/μl) forward and reverse primers, 2.0 µl (50 ng/µl) genomic DNA and 6.5 µl sterile nuclease free water. Thermocycling conditions for +45T/G consisted of an initial denaturation at 94°C for 5 min, followed by 30 cycles of 30 s at 94°C, 30 s at 60°C and 30 s at 72°C with a final extension at 72°C for 5 min. The resulting 367-bp PCR product for +45T/G was digested with *Sma*I (New England Biolabs, Beverly, MA) at 37°C for 12 hours according to the manufacturer’s protocol. The genotype was defined by the absence (T) or presence (G) of the *Sma*I restriction site. Thermocycling conditions for +276G/T consisted of an initial denaturation at 94°C for 5 min, followed by 30 cycles of 30 s at 94°C, 30 s at 58°C, and 30 s at 72°C with a final extension at 72°C for 5 min. The resulting 504-bp PCR product for +276G/T was digested with *Bsm*I (New England Biolabs, Beverly, MA) at 37°C for 12 hours according to the manufacturer’s protocol. The genotype was defined by the presence (G) or absence (T) of the *Bsm*I restriction site. For genotyping, digested fragments were separated by electrophoresis on 2% agarose gel and visualized by ethidium bromide staining.

**Statistical analysis**

All statistical analyses were performed with the SPSS version 15.0 software (SPSS, Inc., Chicago IL, USA). Continuous variables were expressed as mean±SD and compared using student’s *t*-test. All frequencies were estimated using the gene counting method, and both polymorphisms were tested for Hardy-Weinberg’s equilibrium using the chi-squared test. Categorical variables were presented as total number (percentage) and compared by χ^2^-test. OR and 95% CI were estimated for CAD by logistic regression to assess the relative risk conferred by a particular allele and genotype. The haplotypes distribution in CAD and control groups were estimated according to the two-stage iterative method, named expectation maximization algorithm, using the software SNPStats (http://www bioinfo.iconcologia.net/SNPstats). To determine the association between haplotypes and the risk of CAD, a logistic regression model was used, and the most common haplotype was considered as the reference group. The statistical significance level was *P*<0.05. 

## RESULTS

**Clinical characteristics of studied participants**

Demographic and clinical characteristics of the studied participants are shown in Table 1. Patients with CAD were older than those without CAD and also had higher systolic blood pressure, diastolic blood pressure, fasting plasma glucose, homeostasis model assessment of insulin resistance and insulin levels, and the lower levels of total cholesterol, triglyceride, adiponectin, and HDL-cholesterol.

**Genotype distribution and the association of +45T/G polymorphism in** ***ADIPOQ*** **gene with** **coronary artery disease**

**Fig. 1.**
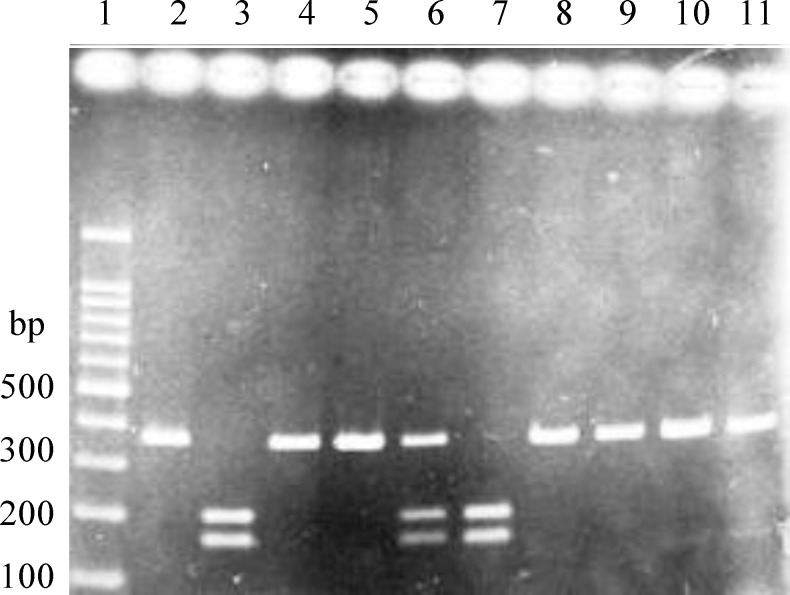
Representative example of PCR-RFLP product for SNP +45T/G. Lane 1, 100 bp DNA ladder; Lanes 2, 4, 5, 8, 9, 10 and 11, wild type TT genotype (367 bp); Lanes 3 and 7, mutant GG genotype (163 bp and 204 bp); Lane 6, heterozygote TG genotype (367 bp, 204 bp, and 163 bp

Polymorphic fragments of the +45T/G were amplified using specific primers, which resulted in a 504-bp product. PCR products were digested with *Sma*I restriction endonuclease into the fragments of 204 bp and 163 bp (GG homozygote), 367 bp, 204 bp, l63 bp (TG heterozygote) as well as 367 bp (TT homozygote) (Fig. 1). The genotype distributions and allele frequency of +45T/G and their corresponding ORs are shown in Tables 2 and 3, respectively. Genotype frequencies of +45T/G were in Hardy-Weinberg equilibrium in both groups. Between two studied groups with and without CAD, the frequency of genotypes for +45T/G was not different (Table 2, χ^2^=3.405, *P*=0.182). Regarding the allele frequency, in CAD cases (87% for T and 13% for G allele) and in controls (80% for T and 20% for G allele), no significant difference was observed between two groups (Table 2, χ^2^=1.961, *P*=0.161). The ORs for CAD was 0.671 (95% CI=0.358-1.257, *P*=0.213) for GT genotype and 0.217 (95% CI=0.024-1.998, *P*=0.176, Table 3) for GG genotype, which were not significant. There was also no significant difference in the genotypes distribution, including TT and TG+GG genotypes of +45T/G between men and women in two groups (*P*>0.05) (Table 4).

**Table 1:** Demographic and clinical characteristics of the studied patients

**Variables**	**Control**	**CAD**	***P*** ** value**
Gender (male/female) (%)	41/59	52/48	0.120
Age (y)	52.20±9.37	57.26±7.42	<0.001
BMI(kg/m^2^)	27.97±4.32	26.62±3.90	0.022
SBP (mmHg)	116.44±24.68	129.54±20.72	<0.001
DBP (mmHg)	72.83±15.77	79.65±14.27	0.003
Total cholesterol (mg/dl)	188.92±62.95	183.49±52.03	0.525
Triglyceride (mg/dl)	184.92±72.25	160.50±74.64	0.023
HDL-C (mg/dl)	46.88±10.35	40.13±8.49	<0.001
LDL-C (mg/dl)	105.05±55.09	111.35±48.96	0.413
Fasting glucose (mg/dl)	158.99±57.75	185.51±67.53	0.003
Fasting insulin (µIU/ml)	14.23±14.11	22.26±21.34	0.009
HOMA-IR	5.87±6.11	10.16±9.83	0.002
Duration of diabetes (y)	4.47±3.12	7.83±3.80	<0.001
Adiponectin (µg/ml)	5.35±2.59	3.45±2.52	<0.001

Data are mean±SD; *P* values for continuous variables were calculated by *t*-test. CAD, coronary artery disease; BMI, body mass index; SBP, systolic blood pressure; DBP, diastolic blood pressure; LDL-C, low-density lipoprotein cholesterol; HDL-C, high-density lipoprotein cholesterol; HOMA-IR, homeostasis model assessment for insulin resistance

**Genotype distribution and association of +276G/T** **polymorphism** **in** ***ADIPOQ*** **gene with coronary artery disease**

**Fig. 2:**
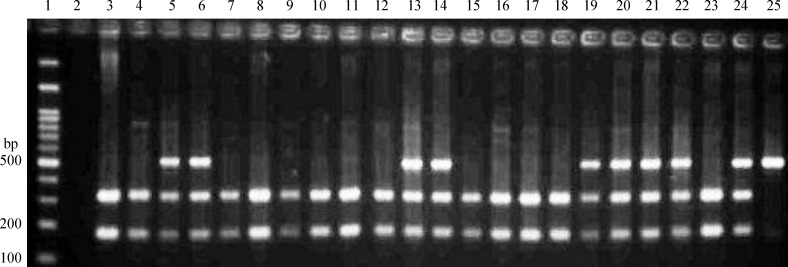
Representative example of PCR-RFLP product for SNP +276G/T. Lane 1, 100 bp DNA ladder; Lane 2, non-template control; lanes 3, 4, 7, 8, 9, 10, 11, 12, 15, 16, 17, 18, and 23, wild-type GG genotype (183 bp and 321 bp); Lanes 5, 6, 13, 14, 19, 20, 21, 22, and 24, heterozygote GT genotype (504 bp, 321 bp, and 183 bp); Lane 25, mutant TT genotype (504 bp

A 504-bp DNA fragment was amplified using specific primers of +276G/T. PCR products were digested with *Bsm*I restriction endonuclease into the fragments of 321 bp and 183 bp (GG homozygote), 504 bp, 321 bp and l 83 bp (GT heterozygote) as well as 504 bp (TT homozygote) (Fig. 2). The genotype distributions and allele frequency of +276G/T and their corresponding ORs are presented in Tables 2 and 3, respectively. Genotype frequencies of +276G/T were in Hardy-Weinberg equilibrium only in T2DM without CAD. The frequency of TT genotype was significantly increased in CAD cases as compared to controls (χ2=7.967, *P*=0.019). A similar significant difference was also found for allele frequency between two groups, which were 66% for G and 34% for T alleles in CAD cases, and 77% for G and 23% for T alleles in controls (χ2=3.895, *P*=0.048) (Table 2). The ORs for CAD were 1.930 (95% CI=1.086-3.43, *P*=0.025) for the GT genotype and 5.158 (95% CI=1.016-26.182, *P*=0.048) for the TT genotype (Table 3), which were significant. There was no significant difference in the distribution of GG, and GT+TT genotypes at +276G/T between men of two groups (χ^2^=2.271, *P*=0.132). Also, there was a significant difference in the distribution of GG, and GT+TT genotypes at +276G/T between women of two groups (χ^2^=4.475, *P*=0.034) (Table 4). 

**Haplotype analysis of two** ***ADIPOQ*** **single nucleotide polymorphisms in association with coronary artery disease**

The haplotype analysis stratified by studied subjects for two loci of *ADIPOQ* gene is shown in Table 5. SNP haplotype frequencies were estimated and compared between diabetic individuals with CAD and without CAD. Based on the four possible *ADIPOQ* haplotypes, the haplotype consisting of wild allele of SNP +276 and mutant allele of SNP +45 was more prevalent in CAD cases compared to the controls (OR=0.37, 95% CI=0.16-0.86, *P*=0.022). The ‘double-mutant’ haplotype (+45G/+276T) was uncommon and was present at very low frequencies in both control and CAD groups. Concerning the association of the haplotype with CAD, our results revealed a significant association between one of the observed common haplotypes (+45G/+276G) and a less risk of CAD in subjects with T2DM (Table 5).

**Table 2:** Genotype distribution and allele frequencies of two studied single nucleotide polymorphisms in all studied subjects

**SNP**		**Genotype frequency (%)**				***P*** a	**Allele frequency (%)**			***P***
+45T/G		TT	TG	GG		0.182	T	G		0.161
Control		65 (65.0)	31(31.0)	4(4.0)			80.0	20.0		
CAD		75 (75.0)	24 (24.0)	1 (1.0)			87.0	13.0		
										
+276G/T		GG	GT	TT		0.019	G	T		0.048
Control		56 (56.0)	42 (42.0)	2 (2.0)			77.0	23.0		
CAD		38 (38.0)	55 (55.0)	7 (2.0)			66.0	34.0		

a Pearson χ2-test; number (% of total); SNP, single nucleotide polymorphism; CAD, coronary artery disease

**Table 3:** The association between genotypes of *ADIPOQ* +45T/G and +276G/T polymorphisms and the risk of coronary artery disease (CAD

**SNP**	**Genotype**	**CAD/control**	**OR (95% CI)**	***P*** ** value**
SNP +45T/G	TT	75/65	1.0	
	TG	24/31	0.671 (0.358-1.257)	0.213
	GG	1/4	0.217 (0.024-1.998)	0.176
SNP +276G/T	GG	38/56	1.0	
	GT	55/42	1.930 (1.086-3.43)	0.025
	TT	7/2	5.158 (1.016-26.182)	0.048

Pearson χ^2^-test was used to determine the genotype distribution; OR, odds ratio; CI, confidence interval

## Discussion

Identification of the association between genetic susceptibility and CAD, particularly in T2DM, has been the subject of many recent studies and offers the potentials for clinical preventive interventions. Although findings about the association between the two common SNPs of *ADIPOQ* gene and many disorders were documented, reports on their association with CAD are rather inconsistent^[^^16^^-^^20^^]^. The present study showed that SNP +276G/T but not SNP +45T/G of the *ADIPOQ* gene was significantly associated with CAD risk in Iranian subjects with T2DM. 

Among more than 10 SNPs of *ADIPOQ* gene reported until now, two common SNPs (+45T/G and +276G/T) were associated with the increased risk of insulin resistance and T2DM in particular^[^^9^^-^^14^^]^. In a case-control study on Italian population, Bacci *et al.*^[^^15^^]^ first reported a significant association between SNP +276G/T and CAD. Recently, a meta-analysis study suggested that the SNP +276G/T of *ADIPOQ* gene is a low risk factor for development of cardiovascular disease in T2DM; however, the association of this polymorphism with the susceptibility to cardiovascular disease in other populations remains unknown^[^^22^^]^. Reports from other studies concerning +276 alleles have shown that T allele of this SNP is associated with higher serum levels of adiponectin and can be considered as a protective factor for diabetes, CAD and hypertension or dyslipidemia in American^[^^23^^]^, Finnish^[^^24^^]^, Japanese, and Korean^[^^25^^]^ populations. However, the results in different populations are somehow debating. 

In the present studied population, we observed that not only GT and TT genotypes but also T allele of SNP +276G/T were related to an increased risk of CAD. Filippi *et al.*^[^^26^^] ^found similar conclusions and reported that T allele of SNP +276G/T was related to premature coronary heart disease in a case-control study in Italian population. They also observed an association between the SNP +276G/T alleles and the serum levels of adiponectin and found that T allele was associated with lower serum levels of adiponectin, which was in contrast with the present study. Moreover, Gui *et al.*^[^^27^^]^ found similar findings and reported that the adiponectin +276G/T was positively correlated with an increased risk of CAD, and the CAD patients had lower levels of adiponectin, which were not affected by different genotypes of +276G/T polymorphism. Conversely, Esteghamati *et al.*^[^^28^^] ^in a study conducted on an Iranian population revealed that T allele of SNP +276G/T in adiponectin gene is significantly associated with a decreased risk of CAD in T2DM patients, which is inconsistent with our results. This discrepancy may be due to the sample size, statistical analysis methods or ethnical background of the studied population. In addition, their study was performed on a relatively homogeneous Iranian population, whereas our study was carried out in a relatively heterogeneous Iranian population composing of different ethnics, including Arabs, Bakhtiaries, and Persians. Thus, ethnic and regional variations may contribute to the observed differences between these studies. Recently, in a case-control study conducted on a Taiwanese population, a significant association was observed between +276G/T polymorphism and T2DM, and T allele was identified as a risk factor for the prevalence of T2DM. In addition, the genotype frequency of SNP +45T/G revealed no difference between T2DM and controls^[^^29^^]^.

**Table 4:** Genotype distributions of +45T/G and +276G/T in *ADIPOQ* gene based on gender

**SNP**	**Genotype**		**Males**				**Females**		
			**CAD/control**	**Χ** ^2^	***P***		**CAD/control**	**Χ** ^2^	***P***
+45T/G	TT		38/28	0.255	0.614		37/38	2.029	0.154
	TG+GG		14/13				11/13		
									
+276G/T	GG		21/23	2.271	0.132		17/33	4.475	0.034
	GT+TT		31/18				31/26		

Pearson's *t*-test was used to determine the genotype distribution

On the other hand, we did not find any significant association between the SNP +45T/G and CAD in T2DM patient, this result is consistent with the reports of several previous studies^[^^10^^,^^15^^,^^17^^,^^28^^]^. In a study on Iranian population, Esteghamati *et al.*^[^^28^^]^ have shown that +45T/G is not associated with the presence of CAD in T2DM patients, which was in consistent with our finding. In contrast, Sabouri *et al.*^[^^30^^]^ study on other Iranian population with CAD indicated that the presence of the G allele of +45T/G may be associated with the risk of CAD. The population of their study consisted of only CAD patients, who had several differences in the clinical baseline characteristics, while our studied population included T2DM patients with CAD, which may affect the reported results. Consistent with our findings, Jung *et al.*^[^^10^^]^ have reported that SNP +45T/G in adiponectin gene is not associated with the presence of CAD. They also found no significant association between the severity of CAD and SNP similar to our findings. Conversely, one study on European population has shown that the G allele of the +45T/G polymorphism is associated with the lower risk of CAD^[^^14^^]^. The protective effect of the G allele was also found in T2DM patients^[^^23^^]^. However, numerous studies have reported contradictory results in the general population or in T2DM patients^[^^5^^,^^9^^,^^10^^,^^26^^-^^31^^]^. It is speculated that the discrepancy might be due to differences in disease definition and ethnic background. 

Our findings indicated that CAD patients had more lower serum levels of adiponectin than control subjects, which were similar to our previous results^[^^20^^]^. Also, another investigation reported an association between plasma adiponectin levels and CAD^[^^32^^]^. We also found that the different genotypes of two common SNPs of *ADIPOQ* gene were not significantly related to the serum levels of adiponectin. These results were obtained and reported in other studies^[^^19^^,^^33^^]^. Nevertheless, the association between *ADIPOQ* SNPs and plasma adiponectin level has been confirmed in some^[^^23^^-^^25^^]^ but not all studies^[^^27^^,^^33^^,^^34^^]^. Recently, the results of a population-based study conducted in South India have revealed that SNP +276G/T was significantly associated with lower serum adiponectin level^[^^35^^]^. Several previous *ADIPOQ* association studies have failed to repeat similar results or have even reported opposite effects of alleles on the adiponectin level. One possible explanation is that *ADIPOQ* gene does not have a key role in the regulation of plasma adiponectin level. On the contrary, several studies have indicated that serum adiponectin levels are highly heritable (30-93%)in different populations^[^^23^^,^^36^^,^^37^^]^. A number of genome-wide scans have failed to demonstrate the association between the adiponectin level and *ADIOPQ *poly-morphisms^[^^10^^,^^27^^,^^33^^,^^34^^,^^37^^]^. Also, where the association was observed, there is a possibility of a linkage between polymorphisms and another mutation in the other genes close to the *ADIPOQ* gene^[^^12^^,^^16^^]^. Moreover, it could possibly be understood because the +276G/T is located in intron 2 and might not affect the serum adiponectin level. 

**Table 5:** The estimation of haplotype frequencies and haplotype association with risk of CAD

**Haplotype**	**Control**	**CAD**	**OR (95% CI)**	***P*** ** value** a
Common haplotypes				
+45T/+276G	0.594	0.588	1.00	
+45T/+276T	0.211	0.281	1.54 (0.86-2.74)	0.150
+45G/+276G	0.176	0.066	0.37 (0.16-0.86)	0.022
				
Uncommon haplotypes				
+45G/+276T	0.019	0.063	4.91 (0.56-42.70)	0.150

a Haplotype frequency determined by the maximum likelihood method. CAD, coronary artery disease; OR, odds ratio; CI, confidence interval.

In the present study, we used haplotype analysis to determine the association between two common SNPs of * ADIPOQ* locus and the risk of CAD in T2DM

subjects. Among four haplotypes, the “double mutant” 45G-276T haplotype was less present in both groups. The haplotype 45G-276G indicated a protective effect against the presence of CAD in T2DM patients as compared to the reference 45T-276G haplotype. Similarly, Esteghamati *et al.*^[^^28^^]^ found that the two haplotypes of adiponectin gene, 45T-276T and 45G-276T, are associated with a decreased risk of CAD in Iranian population. The novel finding of our study is that T2DM patients, who have T allele at position +276 of the *ADIPOQ* gene, probably have a higher risk of CAD than those who carrying G allele.

 In conclusion, our study demonstrated that +276G/T, rather than +45T/G of *ADIPOQ* gene, is more associated with the risk of CAD in T2DM patients. Moreover, one haplotype of these two SNPs had protective effect on the risk of CAD in T2DM patients. The frequency of T allele at position +276G/T in female CAD patients was higher than that of control subjects. Furthermore, females carrying the T allele at position +276G/T had a greater risk of CAD than G allele carriers. These findings suggest that genetic factors may exert a greater influence on CAD in women than in men with T2DM. 
